# A Method for Evaluating Physical Activity Programs in Schools

**DOI:** 10.5888/pcd14.160607

**Published:** 2017-09-14

**Authors:** Cheryl Kelly, Dick Carpenter, Elizabeth Tucker, Carmen Luna, John Donovan, Timothy K. Behrens

**Affiliations:** 1 Institute for Health Research, Kaiser Permanente Colorado, Denver, Colorado; 2 College of Education, University of Colorado–Colorado Springs, Colorado Springs, Colorado; 3 Department of Health Sciences, Northern Arizona University, Flagstaff, Arizona

## Abstract

Providing opportunities for students to be physically active during the school day leads to increased academic performance, better focus, and fewer behavioral problems. As schools begin to incorporate more physical activity programming into the school day, evaluators need methods to measure how much physical activity students are being offered through this programming. Because classroom-based physical activity is often offered in 3-minute to 5-minute bouts at various times of the day, depending on the teachers’ time to incorporate it, it is a challenge to evaluate this activity. This article describes a method to estimate the number of physical activity minutes provided before, during, and after school. The web-based tool can be used to gather data cost-effectively from a large number of schools. Strategies to increase teacher response rates and assess intensity of activity should be explored.

## Background

Health-related factors, including low levels of physical activity, can lead to poor school performance for children (1,2). Conversely, children who are physically active have higher test scores, improved attention, decreased absenteeism, better behavior, decreased stress, and improved mood than children who are not physically active (2). Because of this evidence, Colorado passed House Bill 11–1069 in 2011, which became effective in the 2011–2012 academic year (3). This measure requires all public elementary schools to provide students with a minimum of 600 minutes of physical activity per month (or 30 minutes per school day).

Models such as the Comprehensive School Physical Activity Program have identified ways that schools can incorporate physical activity into the school day, including offering quality physical education (PE) at regular intervals, providing recess, implementing classroom physical activity breaks, or offering before and after school physical activity programming (4).

Many funders are supporting school districts to incorporate more physical activity for students than has been offered. Funders often require districts to work with a contracted external evaluation team to assess changes in the number of physical activity minutes provided to students. Evaluators need methods to estimate how much physical activity students are being offered. Physical activity assessment can occur through 2 methods: 1) indirect methods, which are surrogate markers of physical activity, such as body composition, cardiorespiratory fitness, and surveys or questionnaires, and 2) direct methods, which reflect actual bodily movement or energy expenditure (5) (eg, direct calorimetry, doubly labeled water, motion detectors). Previous studies evaluating physical activity used obtrusive methods such as asking students to wear an accelerometer or having an evaluator observe the class to document when and how much physical activity is offered (6–8). Although these methods provide reliable data, they are expensive and challenging for teachers, and they may lead to social desirability bias (ie, teachers or students may perform differently when being observed than they would when not being observed). Likewise, indirect methods of assessing physical activity, such as self-report, have such limitations as a dependence on recall, a lack of precision about identifying the activity being recalled, inconsistent scoring systems for estimating energy expenditure, and the general overestimation of self-reported physical activity (1,9). We posit that using a method that includes a web-based monitoring tool, whereby teachers and school health coordinators systematically track and report physical activity, may be the best solution for large-scale data collection because of its ability to yield large amounts of data at a reasonable cost. This article describes an evaluation method developed and implemented in school districts funded to increase physical activity opportunities before, during, and after school.

## Data Collection Methods

Twenty-six Colorado school districts that demonstrated a need for more physical activity programming were funded by Kaiser Permanente Colorado. The geographically dispersed schools were funded to implement physical activity before, during, and after school. These districts were asked to select 2 or 3 schools for evaluation. An external evaluation team worked with selected schools to assess whether the number of physical activity minutes offered to students increased during the 2014–2015, 2015–2016, and 2016–2017 school years. To measure physical activity, the evaluation team developed a method to estimate the average number minutes of physical activity per school day per school.

**Classroom-based physical activity.** Classroom-based physical activity is often offered in 3-minute to 5-minute bouts at various times of the day, depending on the teachers’ time to incorporate it, and can comprise various strategies (eg, brain breaks, walking classroom). Because of this variability in how and when classroom-based physical activity is implemented, it is a challenge to evaluate. To track classroom-based physical activity provided by teachers, we developed a web-based monitoring tool that allowed teachers to self-report when they provided a physical activity opportunity and the number of minutes it was provided (Figure). Teachers were asked to report each instance of physical activity they provided during class time (ie, not recess or PE), the grade level of the students, the number of students, the type of activity provided, and the number of minutes each instance was provided.

**Figure:**
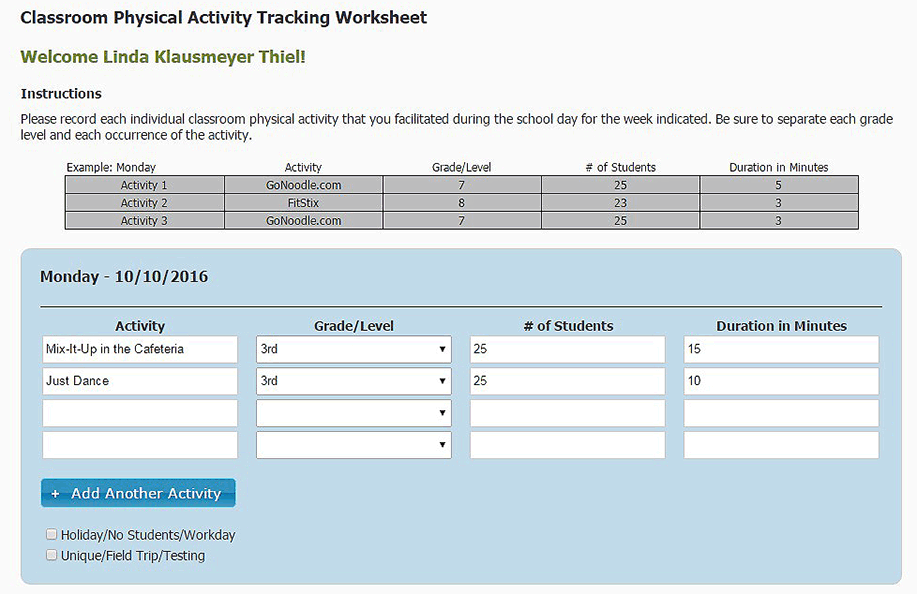
Screenshot of classroom-based physical activity tool.

**Before and after school physical activity programming.** We developed a second component for the web-based monitoring tool to allow each school district’s school health coordinator to report all before and after school physical activity programming provided. Each semester, coordinators reported all programming, the type of program, the number of days it was offered a week, the number of minutes it was offered, and the number of students who participated.

**PE and physical activity during recess. **PE and recess schedules for selected schools were submitted to the evaluation team annually. Using each school’s calendar of holidays, scheduled days off, and the master schedule of PE, we calculated the total number of days that each grade attended PE throughout the year. Total number of recess minutes was calculated as the number of minutes of recess per day times the number of school days.

## Implementation of the Method

The web-based monitoring tool was designed to randomly select teachers 2 or 3 times per semester to report any classroom physical activity they provided during a 1-week period. We sampled teachers weekly by using a stratified random sampling approach where 20% to 30% of each school’s teaching population was selected to participate. The stratified random selection allowed the evaluators to collect data for every week of the semester in each school without burdening every teacher with reporting minutes every week.

Through an automated email delivery system, teachers received an email on Friday morning indicating that the following week was their week to report physical activity minutes. The email included a link to the web-based tool as well as a form to print and track minutes on paper and report all minutes at the end of the day or week. Teachers received an additional reminder mid-week and a final reminder on Friday. In the first year, 35 teachers were invited to participate; in the second year, 43 teachers, and in the third year, 43 teachers. The average response rate in year 1 was low at 14% (range, 1%–49%). In subsequent years, however, the response rates increased steadily, to 27% (range, 4%–68%) in year 2 and 31% (range, 1%–68%) in year 3. The response rate for year 3 was significantly higher than the response rate for year 1 (*F* = 8.07, *P* = .01).

Additionally, each semester, the school health coordinators received a link to the web-based tool and were asked to report all before and after school physical activity programming provided at the selected schools. Similarly, the coordinator was responsible for collecting the recess and PE schedules for each selected school and submitting them by the end of each academic year.

## Data Analysis

To estimate the average number of minutes of physical activity provided to students per year per school, we analyzed data by classroom-based physical activity opportunities, by before and after school programming, and by PE and recess. 

Data on classroom PA were collected at the opportunity level (ie, each instance of physical activity provided during class time and the number of minutes spent in each activity), but our objective was to estimate the number of physical activity minutes provided at the school level. Therefore, these PA minutes were aggregated at the school level by 1) summing each teacher’s daily classroom physical activity minutes and then 2) deriving a mean of all teachers per day per school. The estimates of each school’s minutes per day were then summed over the entire year.

Data on minutes spent in before and after school programming and physical activity during PE and recess were originally collected at the school level; these data required little aggregation. Using each school’s calendar of holidays, scheduled days off, and the master schedule of PE, we calculated the total number of days that each grade attended PE throughout the year (which is not the same as number of school days, because PE is not offered daily and recess ordinarily is). By multiplying the minutes of PE reported by the number of days the class was held, we determined the total number of PE minutes for the year. This total was divided by the total number of school days to provide an average number of PE minutes offered per school day. Similarly, data on the total number of minutes of recess scheduled each day of the week in each school were provided by the district’s school health coordinator and used to calculate the total number of recess minutes offered to students during the year.

Total before and after school minutes is the number of minutes of before and after school programming times the number of days it was offered. When multiple programs were offered on the same day, we used the mean of the minutes per school in the calculation.

The total number of minutes is the sum of minutes spent in classroom opportunities, before and after school programming, PE, and recess. This total number of minutes provided the estimate for the average number of physical activity minutes provided by each school. Additionally, to assess the number of physical activity minutes provided per day on average, the total number of physical activity minutes per year was divided by number of school days to estimate the overall average number of physical activity minutes offered per day.

These analyses allowed the evaluator to estimate and track the average number of minutes provided during class time, before and after school programming, and during PE and recess. The evaluation team shared each school’s results and response rates with each coordinator yearly. The funder provided technical assistance and professional development opportunities to the coordinators, so they could identify opportunities for increasing minutes at certain times of the day or identify strategies that seem to be working well and could be replicated in other schools.

## Limitations of the Method

Like any method of data collection, our method has limitations. Our web-based tool relies on self-report, which has limitations. However, many limitations of self-report are observed at the individual level, for example, when people report their own physical activity. The extent to which these limitations apply to reports on the physical activity of others is not known.

A second limitation of our data collection method is the response rate. The response rate found among teachers in our study is not dissimilar to rates reported in other research, and it is likely an accurate reflection of the difficulty of collecting data from teachers. Achieving response rates anywhere close to 100% is typically extremely difficult or prohibitively expensive (10). It may be tempting to dismiss our data collection system because of our response rates, but we believe this to be ill-advised. Despite the response rate, our data collection system has distinct advantages over other methods, such as direct observation or paper teacher logs. Compared with other methods, our electronic method allows for quicker responses, more graphically interesting surveys, the use of many more response tools, lower costs, and greater flexibility; these advantages of electronic data collection have been discussed (11,12). Moreover, the response rate may be an important diagnostic and evaluative indicator, showing that teachers simply may not have perceived the initiative, or at least the reporting of physical activity opportunities, as important, interesting, or relevant. This observation would be entirely consistent with other research indicating the difficulties associated with altering teacher practices when introducing new programs or interventions (13). Viewed in this way, the low response rate may not entirely be a limitation.

Another limitation is that we did not ask teachers or coordinators to report the intensity of physical activity. Although intensity is not a factor in assessing the number of physical activity opportunities, it is critical to estimating energy expenditure. Thus, it would be imperative that a metric for determining physical activity intensity be included if the objective of the evaluation is to measure energy expenditure.

## Implications

The value of our method of data collection is the ability to gather data cost-effectively from a large number of schools. In particular, it is a way for every school to be represented every week. Response rate is not the only construct of interest; so too is representation (14). Because the number of schools was large, traditional means of collecting data — direct observation, tracking devices, and the like — were not financially feasible. We could have taken a random sample of schools, but our methods resulted in arguably greater representation than would a random sample, because every school was represented every week.

To strengthen this method, we recommend validating teacher reports by collecting data simultaneously via accelerometers or other such devices, observing directly, or video recording classroom activities, just to name 3 examples. Similarly, it would be instructive to measure the extent to which the classroom-based physical activity of teachers who do not respond differs from the classroom-based physical activity of teachers who do respond to the program. If differences are small, then they may not be of great concern.

As mentioned above, the low response rate may be a measure of something important, particularly if traditional methods of increasing response rates have been taken. We recommend pursuing ways of increasing response, including one we were unable to afford — incentives. Doing so could be seen as a win–win. Even if incentives fail to increase response rates, they may tell us something essential about how participants view the relevance or importance of the intervention.

Finally, patterns of physical activity during the school year suggest the method of data collection accurately captured variation in physical activity. For example, because data were collected weekly, we were able to compare levels of physical activity by semester. Levels of physical activity in the second semester (spring) were typically lower than levels in the first semester (fall), which we expected. Because of extensive school, district, and state testing and inclement weather, opportunities for physical activity in the spring were fewer than those in the fall.

## Lessons Learned

Tools for monitoring physical activity need to be as specific, consistent, and closed-ended as possible. During the first year of data collection, we used open-ended response fields that required many hours of cleaning and coding by the evaluation team. We fixed this in year 2 and year 3 by maximizing the use of closed-ended functions.

Additionally, on the basis of feedback from the school health coordinators indicating that teachers are busy and have many requirements during the school day, we began sending a weekly email to each district school health coordinator that listed the teachers selected to participate that week. The school health coordinators could then personally remind each teacher to participate, provide them with a paper reporting form if necessary, and follow up with any questions. These personal reminders probably increased our response rates in the second and third years, because someone from the school system, rather than an outside entity, was reaching out and encouraging participation.

Overall, this web-based monitoring tool can be used to evaluate changes in physical activity programming in schools. Implementing a tool like the one described here would allow teachers and school health coordinators to systematically track and report physical activity. The tool may be the best bet for large-scale data collection because of its ability to yield large amounts of data at a reasonable cost.
